# A Urinary Bcl-2 Surface Acoustic Wave Biosensor for Early Ovarian Cancer Detection

**DOI:** 10.3390/s120607423

**Published:** 2012-05-31

**Authors:** Onursal Onen, Alper Sisman, Nathan D. Gallant, Patricia Kruk, Rasim Guldiken

**Affiliations:** 1 Department of Mechanical Engineering, University of South Florida, 4202 E. Fowler Avenue, ENB 118, Tampa, FL 33620, USA; E-Mails: onursalonen@mail.usf.edu (O.O.); alper.sisman@marmara.edu.tr (A.S.); ngallant@usf.edu (N.D.G.); 2 Department of Electrical and Electronics Engineering, Marmara University, Goztepe 34722, Istanbul, Turkey; 3 Department of Pathology and Cell Biology, University of South Florida Health, 12901 Bruce B. Downs Blvd., MDC 11, Tampa, FL 33612, USA; E-Mail: pkruk@health.usf.edu

**Keywords:** ovarian cancer, MEMS, piezoelectric, sensor, early detection, Bcl-2, SH, SAW, SAM, ultrasound

## Abstract

In this study, the design, fabrication, surface functionalization and experimental characterization of an ultrasonic MEMS biosensor for urinary anti-apoptotic protein B-cell lymphoma 2 (Bcl-2) detection with sub ng/mL sensitivity is presented. It was previously shown that urinary Bcl-2 levels are reliably elevated during early and late stages of ovarian cancer. Our biosensor uses shear horizontal (SH) surface acoustic waves (SAWs) on surface functionalized ST-cut Quartz to quantify the mass loading change by protein adhesion to the delay path. SH-SAWs were generated and received by a pair of micro-fabricated interdigital transducers (IDTs) separated by a judiciously designed delay path. The delay path was surface-functionalized with monoclonal antibodies, ODMS, Protein A/G and Pluronic F127 for optimal Bcl-2 capture with minimal non-specific adsorption. Bcl-2 concentrations were quantified by the resulting resonance frequency shift detected by a custom designed resonator circuit. The target sensitivity for diagnosis and identifying the stage of ovarian cancer was successfully achieved with demonstrated Bcl-2 detection capability of 500 pg/mL. It was also shown that resonance frequency shift increases linearly with increasing Bcl-2 concentration.

## 1. Introduction

Ovarian cancer is the fifth leading cause of cancer death among women in United States and it has a 1.4% (1 in 71) lifetime risk [1]. Diagnosis of ovarian cancer in the early stages currently accounts for only 30% of all cases, and in most late stages the cancer is lethal. The lack of overt symptoms and the absence of a reliable screening test results in over 70% diagnoses occurring the disease has spread beyond the ovary, so the prognosis is poor [1]. The 5 year survival rate after diagnosis for late stage disease is less than 40%. Currently, pelvic examination, ultrasound and blood levels of serum biomarker CA125 are the standard screening methods for ovarian cancer [2–4]. However, each of these ovarian cancer detection methods has limitations. Pelvic examination is known to be obstructed by the intraperitoneal location of the ovaries and is typically capable of late-stage disease detection only. Similarly, ultrasonic examination does not possess the capability of distinguishing between benign and malignant cases and is subject to variation in interpretations among sonographers. CA125 is the current standard biomarker for ovarian cancer diagnosis and monitoring [4]. It is present in the blood serum of ovarian cancer patients. However, it has been shown that CA125 levels can also be elevated due to other disorders, including inflammation, benign gynecological disease, or hepatic disease, leading to false positive results [5,6]. There are other biomarkers that have been associated with ovarian cancer such as eosinophil-derived neurotoxin [7], Mesothelin [8], VEGF [9], and HE4 [10]. There also exists a few biochips relying on fluorescence or chemiluminescence for ovarian cancer monitoring based on DNA sequences (testing for ovarian cancer-related mutations) [11,12] and protein biomarkers [13,14]. However, these biosensors use complex reagents such as DNA extraction kits and expensive laboratory equipment including fluorescence microscopes or plate readers, thus, are not suitable for point-of-care testing [15]. Recently, an enzyme-linked immunosorbent assay (ELISA) test based on a cell-phone-coupled optical sensor has been presented for point-of-care quantification of urinary HE4 levels [15]. However, the chemicals and substances used during ELISA tests are still fairly expensive, and special attention should be given for storage. The absence of reliable screening methods to detect early ovarian cancer contributes to poor prognosis. Therefore, the development of a new, reliable, simple, safe, and economic testing platform to detect ovarian cancer is imperative.

Bcl-2 (B-cell lymphoma 2) is a protein that is directly related with apoptosis of healthy and cancer cells [16]. Apoptosis is the most common form of programmed cell death (cellular autophagocytosis, anoikis and necrosis are other forms). It has several other crucial functions, such as formation of the embryo, tissue maintenance, cellular homeostasis, terminating immune responses, and restricting the spreading of infections [17]. The Bcl-2 family, named after the Bcl-2 protein itself, includes both anti-apoptic and pro-apoptic constituents that control the release of catalysts of cell death. It was previously shown that urinary Bcl-2 levels are elevated during different stages of ovarian cancer [18,19]. Clinical validation of urinary Bcl-2 as a reliable biomarker for ovarian cancer was conducted with ELISA tests using urine samples collected from 388 patients, including healthy controls and patients with benign gynecological disorders, early- and late-stage ovarian cancer [18]. The average urinary level of Bcl-2 was found to be 0.59 ng/mL in healthy controls, 1.12 ng/mL in benign disorders, 2.60 ng/mL in early-stage ovarian cancer and 3.58 ng/mL in late-stage ovarian cancer. The highest Bcl-2 concentration observed in the study was around 10 ng/mL. The number of samples, average concentration, and standard deviation of Bcl-2 for these four patient groups are listed in Table 1. Signs of poor early stage diagnosis can be observed from the table of samples included in this study which represents actual availability of samples from tissue banks containing each stage of samples. Thus, analyzing the values in Table 1, the minimum detectable target concentration of Bcl-2 was chosen to be 0.5 ng/mL for design and for experimental characterization studies reported herein.

The efficacy of Bcl-2 as a biomarker for ovarian cancer was further validated by comparison to CA125 serum levels using ELISA tests on 35 samples from the same cohort [18]. The comparison of Bcl-2 and CA125 levels for the same samples shows efficacy of Bcl-2 as a urinary ovarian cancer biomarker for reliable dual screening with CA125.

**Figure 1.**
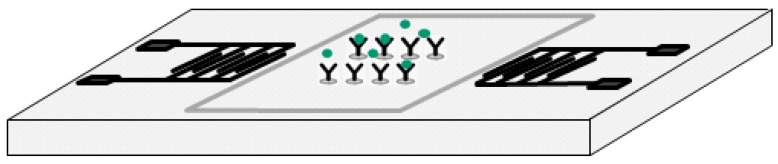
Illustration of the sensor.

The studied biosensor employs shear horizontal (SH) surface acoustic waves (SAWs) to identify mass loading changes caused by Bcl-2 binding specifically to antibodies on the sensor surface. It is composed of a pair of interdigital transducers (IDTs) microfabricated on ST-cut Quartz wafers in the direction 90° off x-axis and delay path specifically functionalized to capture Bcl-2 proteins while minimizing non-specific adsorption (Figure 1). An experimentally-verified optimized surface functionalization scheme was employed for effective capture of Bcl-2 protein while maximizing sensitivity and selectivity. The developed surface functionalization technique also minimizes the non-specific binding to the sensor surface. The sensor's electrical connections were made by low-conductivity silver-reinforced epoxy. The experimental characterizations of the sensor's response to varying Bcl-2 concentrations were performed in a custom-designed oscillatory circuit. The oscillatory circuit was composed of two RF amplifiers connected in series, a frequency counter, an oscilloscope (to monitor the signal), and the sensor, which was used as the feedback element determining the oscillation frequency. The characterization was performed by using multiple sensors with up to 10 tests conducted on a sensor by cleaning the delay path with 1.5 M NaCl solution in de-ionized (DI) water. The tests were done by placing 80 μL droplets of Bcl-2 solutions (in Dulbecco's phosphate-buffered saline—DPBS) with various concentrations on the delay path. Quantification of the Bcl-2 concentration was achieved by monitoring the frequency shift for each solution. The frequency shift was caused by the change in surface density of the delay path (mass loading). As surface density increased by protein adhesion, SAW velocity decreased, resulting in a reduction in the oscillation frequency that was measured by the frequency counter. The frequency shift for each tested concentration was measured, and the sensor was successful in detecting Bcl-2 concentrations as low as the target concentration, 0.5 ng/mL. It was observed that the frequency shift had a linear trend corresponding to increasing Bcl-2 concentration. Additionally, minimal frequency shift was observed for the control DPBS solution with no Bcl-2 present.

In the following section, important design parameters, fabrication of the sensor, and surface functionalization are discussed in detail. In Section 3, the electrical characterization of the sensor and results are presented. The final section covers the discussions and conclusion along with the future work.

## 2. Sensor Design and Fabrication

### 2.1. Sensor Design

The sensor uses shear horizontal surface acoustic waves, which are frequently used for liquid-loaded biosensing applications. In SH-SAWs, the particle displacement is in the plane of the surface (unlike normal-to-surface displacement of Rayleigh waves). SH-SAWs are not affected or damped by liquid loading, as compared to Rayleigh waves, in which the particle displacement is directly coupled with the liquid on top and highly damped by mass loading of the liquid itself. Thus, Rayleigh waves are virtually insensitive to mass loading changes in liquid sensing applications. On the other hand, almost all SH wave propagation on various substrates results in leaky waves (not pure waves like Rayleigh waves), which also leak into longitudinal and shear vertical wave components when excited. For this reason, special cuts of typical wafer types of wafers are typically used for SH waves, in which the energy is highly concentrated on the SH mode. Typical wafer types used in SH-SAWs are ST cut Quartz, 41° and 36° Y-cut lithium niobate (LiNbO_3_), and 36° Y-cut lithium tantalate (LiTaO_3_). The sensing mechanism of SH-SAW sensors relies on the change of SAW speed either by change in mass loading (most biological and chemical sensors) or by changing physical parameters. In general, the majority of SAW sensors include surface treatments and extra layers to effectively and specifically sense the target analyte. Several SH-SAW sensors have been reported using 36° Y-cut LiTaO_3_ [20], ST-cut Quartz [21,22], 36° Y-Cut Quartz [23], 41° Y-cut LiNbO_3_ [24,25], 64° Y-cut LiNbO_3_ [26,27], potassium niobate (KNbO_3_) [28], and langasite [29]. In the design stage, different alternative SH-SAW generating wafers (such as ST-cut Quartz, 36° Y-Cut LiNbO_3_, and LiTaO_3_) were tested with identical delay path designs and surface functionalization steps. It was observed that ST-cut Quartz is the most stable and the easiest to operate among those tested. ST-cut Quartz is also favorable for narrower bandwidth operation, and it does not need additional layers or gratings to concentrate the energy in the surface. SH waves are present in the direction of 90° off the x-axis (parallel to primary flat) in ST-cut Quartz, so the features were designed to obtain wave propagation in this particular direction.

The substrates used in this study were 3-inch, single-side-polished, 500 μm-thick ST-cut Quartz wafers. The SH-SAWs were generated and sensed by a pair of interdigital transducers separated with a delay path on these wafers. The pitch (corresponding to the wavelength of the SAW) was chosen as 300 μm, ensuring fabrication yield and tolerable wave attenuation through the delay path. Each finger of the IDT was 75 μm wide (corresponding to the one quarter of wavelength) for the most efficient SAW generation, as reported by others [30]. The design parameters of the sensor are illustrated in Table 2.

### 2.2. Fabrication

**Figure 2.**
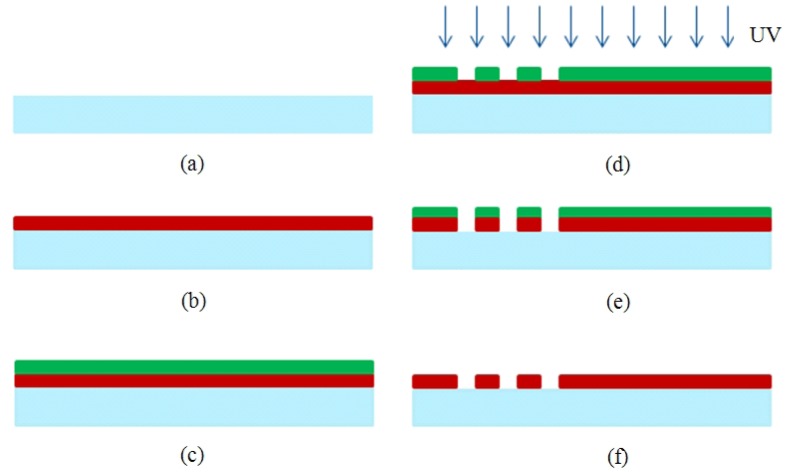
Overview of sensor fabrication: (**a**) ultrasound cleaning; (**b**) chromium deposition; (**c**) photoresist spinning; (**d**) exposure; (**e**) wet etching of chrome; (**f**) photoresist stripping.

**Figure 3.**
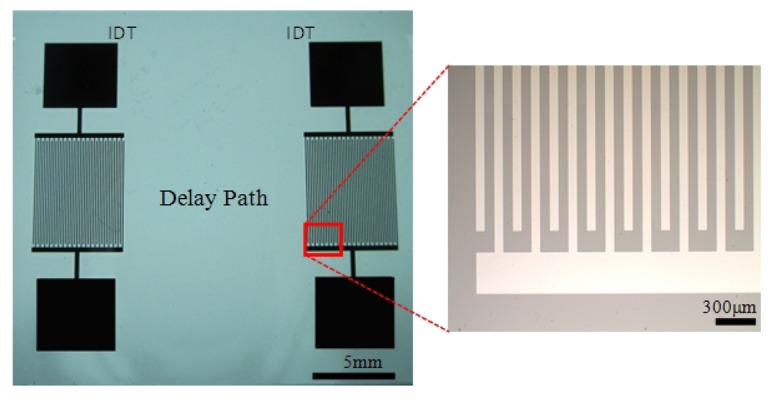
Fabricated ST-cut Quartz sensor.

The IDTs were microfabricated using conventional MEMS fabrication techniques with a single-mask photolithography process. The fabrication steps are illustrated in Figure 2. The fabrication process started with cleaning the wafer in an acetone bath placed in an ultrasound cleaner for 10 min, followed by rinsing with methanol and DI water and dried by a stream of nitrogen (Figure 2(a)). The metal film (Cr) was then sputtered by DC sputtering (CRC Sputter, Torr International, New Windsor, NY, USA) for 5 minutes at 200 mA constant current to obtain a film thickness of 1,000 Å (Figure 2(b)). After sputtering, the wafers were cleaned once again with acetone, methanol, and DI water and dried with nitrogen. A positive photoresist S1813 (Shipley, Marlborough, MA, USA) was then spun on the wafer. Photoresist was spun initially for 10 seconds at 700 rpm to be spread around the wafer, and then for 40 seconds at 3,000 rpm to reach the desired thickness (Figure 2(c)). A photoresist thickness of 1.6 μm was obtained using this recipe. The wafers were then soft-baked at 90 °C for 60 seconds before exposure. After soft bake, the wafers were exposed to UV light for 5 seconds with an average intensity of 25 W/m^2^ to transfer the features from the mask to the wafers (Figure 2(d)). The exposed wafers were then developed in developer MF-319 (Shipley) for 70 seconds to remove the exposed portions of the photoresist. The wafers were then hard-baked for 5 minutes at 100 °C. The features were formed by etching for 40 seconds (Figure 2(e)). Then, the remaining photoresist was removed in an acetone bath (Figure 2(f)). After completing the fabrication process to realize the sensors, the wafers were coated with photoresist to protect the features during the dicing process. The dicing of the ST Quartz wafer was performed using resin-bonded diamond blades at a spindle speed of 20 K rpm and a feed rate of 1 mm/s in the dicing saw. One of the fabricated ST-cut Quartz sensors used in this study is illustrated in Figure 3. It should be noted that the yield was found to be very close to 100% with these optimized fabrication parameters.

The electrical connections to the sensors were obtained with a low-conductivity silver-reinforced conductive epoxy (Duralco 120, Cotoronics Corp., Brooklyn, NY, USA). This connection method was preferred over the wire bonding method because stronger connections are desired during tests and it is not possible to solder chromium. Unlike the wire bonding method, it was observed that the conducting silver epoxy successfully withstood the solvents used in the surface functionalization of the delay path.

### 2.3. Surface Functionalization

**Figure 4.**
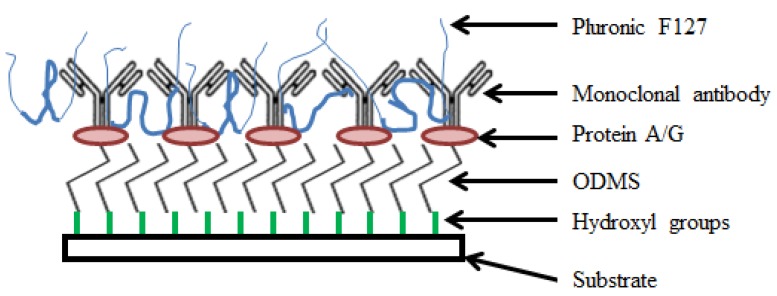
Illustration of surface functionalization.

The sensor will quantify Bcl-2 levels in aqueous solutions, so it is necessary to apply surface treatments to the delay path section of the sensor to specifically and selectively sense the target protein. Molecular self-assembly techniques involving bioconjugation were employed to develop an effective Bcl-2 capture method. Bioconjugation can be defined as molecular linking of two or more components to construct a compound—namely, molecular self-assembly of several reagents for a desired purpose [17,31]. A scheme was developed to isolate the Bcl-2 protein from a solution via specific binding to a monoclonal capture antibody that is immobilized on the sensor surface. More specifically, self-assembled monolayers (SAMs) and blocking agents were used to optimize Bcl-2 capture on the sensor surface. The surface functionalization developed in this study is novel as it is the first of its kind to isolate and capture Bcl-2 protein using functionalization by surface assemblies. The method was optimized for most effective Bcl-2 capture, with trials of several different SAMs and recipes, which were presented in detail in [17]. The optimization was handled by modified sandwich ELISA tests. The surface functionalization recipe employed in this study is summarized in the following steps (Figure 4):

- First, oxygen plasma cleaning of the surface was performed to remove organic residues and form hydroxyl groups on the surface. The sensors were oxygen plasma-cleaned for 5 minutes. The beaker in which the silanization was performed also was plasma-cleaned for 5 minutes. The hydroxyl groups serve as a foundation for organofunctional silanization.
- Silanization was done using the organosilane, octyldimethyl silane (ODMS, molecular formula CH_3_(CH_2_)_7_Si(CH_3_)_2_Cl, Sigma Aldrich, St. Louis, MO, USA) using a solution of 474 μL of stock ODMS in 20 mL of acetone resulting in an ODMS concentration of 0.1 M. ODMS provides the linkage between organic and inorganic domains through hydrophobic interactions. The sensors were kept in the solution for 30 minutes, while IDTs were protected by Kapton tape. If the IDTs were not protected, it was observed that the chromium was attacked by HCl formed during the silanization process, and the sensor did not work properly. After the silanization step, the sensors were rinsed with acetone and ethanol and dried with nitrogen.
- Protein A/G (Pierce Recombinant Protein A/G, Thermo Fisher Scientific, Rockford, IL, USA) with a concentration of 1 μg/mL in Dulbecco's Buffer Phosphate Silane (DPBS, Life Technologies, Grand Island, NY, USA) was employed for immobilizing antibodies. The sensors were again placed completely in the solution for 1 hour. Protein A/G was adsorbed directly on the ODMS to ensure proper orientation of the antibodies on the sensor surface by binding their constant fragment (F_c_) domains. The sensors were rinsed with DPBS after this step.
- Immunoglobin G (IgG) antibodies (polyclonal rabbit anti-human Bcl-2, Sigma Aldrich, St. Louis, MO, USA) were used for Bcl-2 capture, which are Y-shaped with two antigen binding (F_ab_) regions and one F_c_. The F_c_ regions were immobilized with the help of Protein A/G onto the surface, resulting in properly-oriented free F_ab_ regions for the most effective Bcl-2 capture and therefore maximum sensor surface affinity. A working concentration of 5 μg/mL of anti-Bcl-2 in DPBS was placed on the sensor surface as a droplet covering only the delay path for 1 hour. The sensor surface was then rinsed with DPBS before the final step.
- Pluronic F127 (Sigma Aldrich) was adsorbed to obtain a non-fouling surface for highly selective Bcl-2 capture, which is essential for a diagnostically applicable sensor. The Pluronic, a tri-block copolymer whose non-fouling nature is mediated by its two polyethylene glycol (PEG) chains, prevents other molecules from non-specifically attaching to the sensor surface. The sensor was submerged in 10 μg/mL Pluronic F127 in DI water for one hour and then rinsed with DI water.

## 3. Measurement Setup and Results

### 3.1. Oscillatory Circuit Design and Experimental Setup

**Figure 5.**
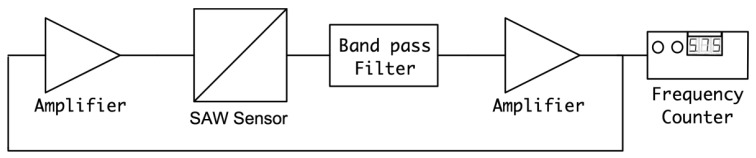
Block schematic for oscillator circuit.

The SH-SAW biosensor was employed in a custom-designed oscillatory circuit for quantifying the Bcl-2 concentrations. An oscillatory circuit configuration was selected due to its higher sensitivity as compared to other detection methods such as vector voltmeter or network analyzer. In the custom oscillator circuit, the sensor was used as the feedback element of the RF amplifier. In this configuration, the relative change in SAW velocity due to Bcl-2 adhesion to the delay path leads to an oscillation frequency shift. This change in oscillation frequency was detected with a digital frequency counter, which was reported to accurately identify acoustic wave velocities [32]. The setup used for Bcl-2 characterization involves the biosensor, two variable gain RF amplifiers (Olympus 5073PR and Olympus 5072PR, Olympus NDT Inc., Waltham, MA, USA), a digital frequency counter (Agilent 53220A, Agilent Technologies Inc, Santa Clara, CA, USA), an oscillator (Tektronix TDS2001C, Textronix Inc., Beaverton, OR, USA) and the specifically-designed analog filter, as illustrated Figure 5. Two RF amplifiers were used to enable optimization of the loop gain [33].

**Figure 6.**
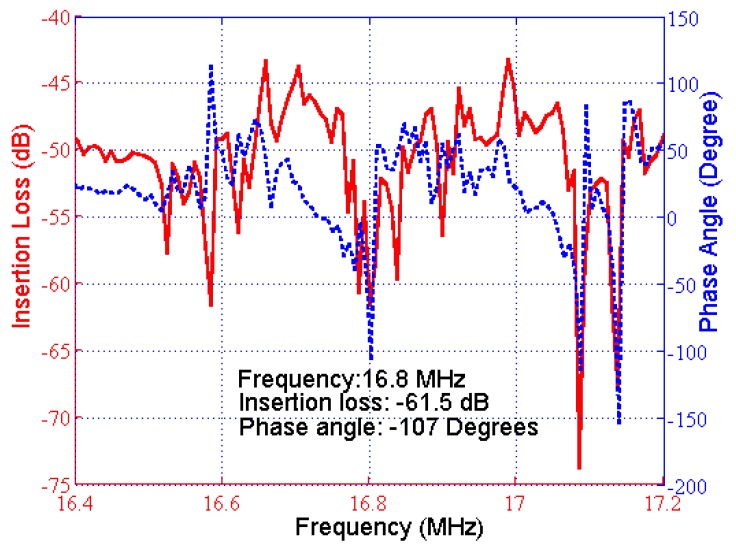
Frequency response of sensor: insertion loss (red, solid), phase (blue, dashed).

As the SH-SAW biosensor was used as a resonator, the frequency response was investigated in order to identify proper circuit satisfying oscillation conditions. Experimentally-measured frequency response is depicted in Figure 6. An insertion loss of 61.5 dB was measured when there was liquid on the functionalized delay line. The frequency response shows several peaks around the designed oscillation frequency (16.8 MHz).

**Figure 7.**
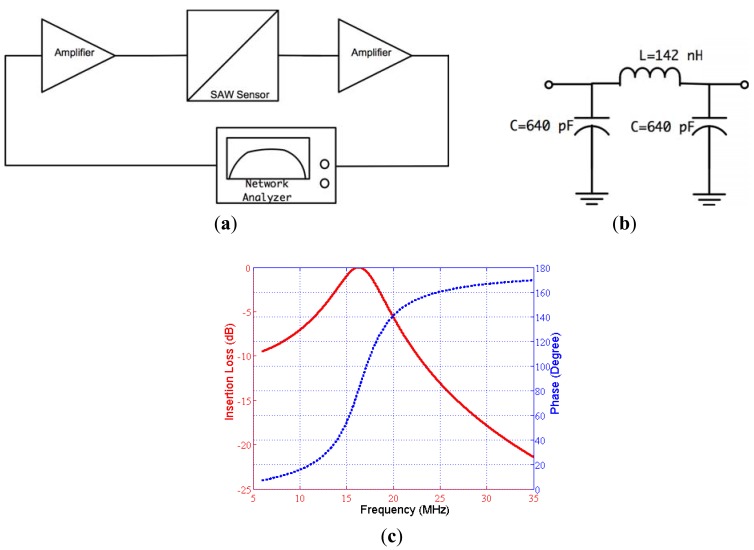
(**a**) Block diagram of loop-gain and loop phase measurement setup; (**b**) designed pi type filter; (**c**) frequency characteristics of filter: insertion loss (red, solid), phase (blue, dashed).

The designed feedback/loop based oscillator had a measured short-term frequency stability of less than 3 ppm. The experiments show that this sensitivity enabled the detection of Bcl-2 concentration as low as 0.5 ng/mL, which was sufficient for this study. The gain of the amplifiers were adjusted, and the passive filter was designed in such a way that the loop gain was 0 dB or higher and the phase shift in the loop was equal to 0°. The oscillation started after these two conditions were met and the exact resonance frequency was the frequency that makes the phase 0°, not the point of peak gain. We chose to use a physical circuit measurement technique rather than simulation techniques for filter design due to the complicated impedance spectrum of SAW devices and the requirement of accurately identifying the total phase in the oscillator loop [34]. The open loop gain and phase were measured by an experimental setup illustrated in Figure 7(a). A network analyzer (Agilent 5061A, Agilent Technologies Inc.) was used to measure open loop gain and phase satisfying the aforementioned oscillation conditions.

The first oscillation condition required adjustment of the amplifier gain to obtain a total loop gain of at least 0 dB, compensating for all of the losses in the loop. The noise figure of the amplifiers was also important since it affected the frequency stability of the oscillator. The insertion loss of the sensor was −61.5 dB (Figure 6); hence, a feasible range of 61 to 65 dB was determined for the sensor. The passive filter also had an insertion loss of 2 dB; hence, a minimum total gain of 67 dB was supplied by RF amplifiers to meet the first oscillation condition.

The second oscillation condition was to make the total loop phase 0° and was satisfied using a passive filter. The designed band-pass filter also helped to eliminate the undesirable peaks in the frequency response of the SAW sensor. A pi-type LC band pass filter was designed with 16.8 MHz center frequency and 30% fractional bandwidth. The phase at the center frequency was determined using the set up illustrated in Figure 7(a). The phase of the filter was designed to obtain 0° total phase in the loop. The designed filter circuit and its frequency characteristics can be seen in Figure 7(b,c). The circuit parameters were determined with the developed MATLAB code then were fine-tuned by the trial and error method [35].

**Figure 8.**
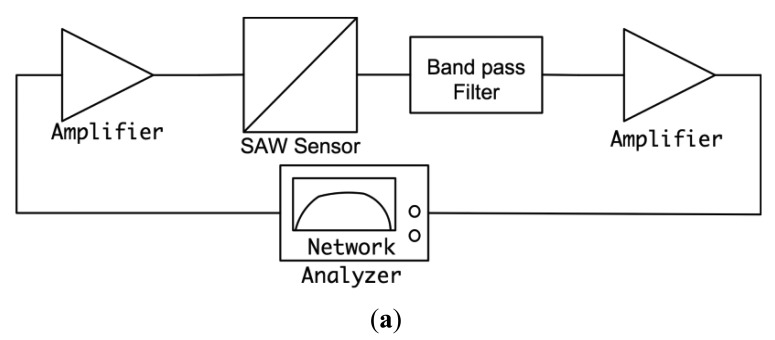

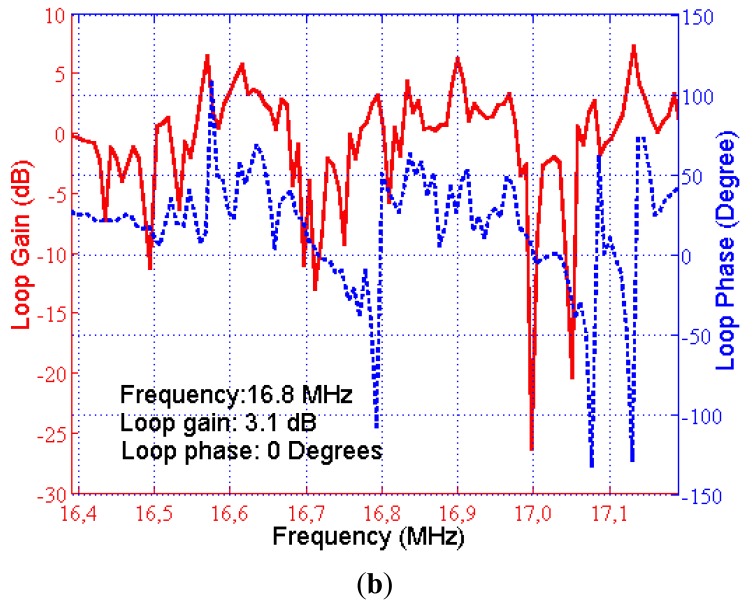
(**a**) Block diagram of loop gain and loop phase measurement setup; (**b**) measured loop gain and loop phase plots: loop gain (red, solid), loop phase (blue, dashed).

The magnitude and phase response of the loop was also measured after insertion of the filter block. The measured open loop phase lag and the loop gain in the required oscillation frequency were found to be 0° and 3.1 dB, respectively. The block diagram of the measurement setup and the results are illustrated in Figure 8(a,b), respectively. As a result, the oscillation conditions were satisfied with this custom designed circuit and oscillation started when the loop was closed in the setup illustrated previously in Figure 5.

### 3.2. Results

The tests were performed using the setup illustrated previously in Figure 5 with Bcl-2 solutions in DPBS ranging from 0.5 to 12 ng/mL. Before undertaking these important tests, each fabricated SAW biosensor was tested using a network analyzer (Agilent 5061A) before and after surface functionalization. Frequency response and phase content of the sensors were recorded and compared with other and virgin sensors ensuring proper operation. Several ST Quartz sensors were tested with droplets of Bcl-2 solutions for 10 minutes to ensure reproducibility and repeatability. Each fabricated and surface functionalized sensor was used in several tests by soaking them in 1.5 M NaCl solution in DI water, rinsing with DI water, and drying them under a nitrogen stream between uses. Each sensor was able to be tested up to 10 times by following this technique to remove Bcl-2 proteins attached to the antibodies. Placed manually on the delay path were 80 μL droplets of Bcl-2 solutions to ensure that the shape and coverage of the droplets on the delay path were same in each test.

**Figure 9.**
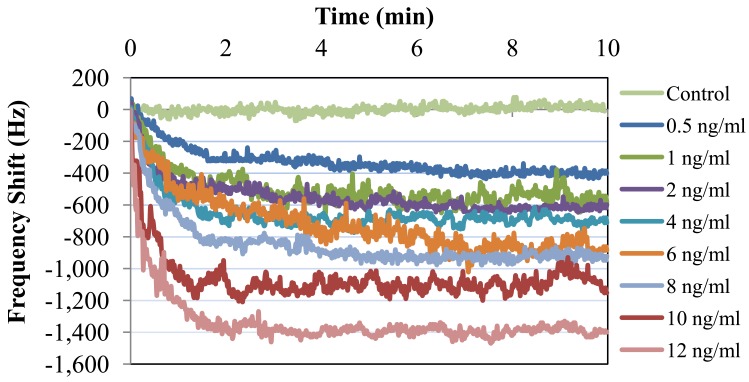
Measured frequency shift (Hz) for various concentrations of Bcl-2 in DPBS.

**Figure 10.**
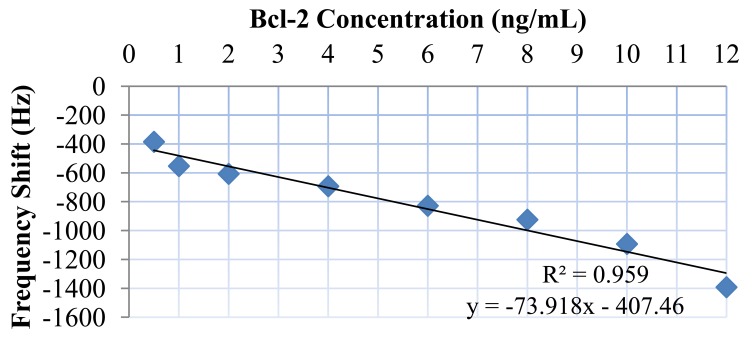
Average frequency shift measured, corresponding to different Bcl-2 concentrations and best line fit (R^2^ = 0.959).

Bcl-2 solutions with concentrations of 0.5, 1, 2, 4, 6, 8, 10, and 12 ng/mL were tested along with a control (DPBS only). Each test sample was monitored for 10 minutes, because no significant shift in frequency was observed after this duration. The results of these tests are presented in Figure 9. As can be observed from the figure, there was a clear linear trend of increasing oscillation frequency shift with increasing Bcl-2 concentration. The control test showed almost no shift in oscillation frequency. It can also be observed from the shape of the curves that the protein adhesion was nearly completed by the end of the second minute. The lowest concentration tested resulted in a frequency shift of >300 Hz, which is a detectable value with an oscillation frequency of 16.8 MHz. In Figure 10, the shift in oscillation frequency is presented as a function of Bcl-2 concentration. The frequency shift values were calculated by taking the average of data after 2.5 minutes. As seen from the figure, the shift in frequency increases linearly with increasing concentration.

## 4. Discussion and Conclusions

In this study, an ultrasonic MEMS biosensor for detection of urinary anti-apoptotic protein Bcl-2 was successfully designed, fabricated, and experimentally characterized. SH-SAWs were employed with microfabricated IDTs on ST-cut Quartz to quantify the Bcl-2 concentration. SH-SAWs were generated and sensed by a pair of micro-fabricated IDTs separated by a surface functionalized delay path. An optimized recipe using SAMs of ODMS, Protein A/G, monoclonal antibodies, and Pluronic F127 was employed for the most effective Bcl-2 capture. The method was optimized for specificity and selectivity, with trials of several different similar SAMs.

The sensor was experimentally characterized in a resonator circuit by placing buffer solutions of Bcl-2 of known concentration (in DPBS) on the delay path. Bcl-2 concentrations were characterized by the resulting resonance frequency shift caused by the mass loading increase of biomarker binding, which reduces the speed of the SH-SAWs. The target sensitivity for diagnosis and quantifying the stage of ovarian cancer was achieved with successful detection of Bcl-2 in the concentration range of 0.5 to 12 ng/mL. It was also observed that there is a linear relationship between the shift in resonance frequency and Bcl-2 concentration. Each sensor was used up to 10 times after applying 1.5 M NaCl solutions in DI water to remove the proteins attached to the antibodies. It was also observed that due to optimized fabrication process, the inter- and intra-array variation was minimal and had no measurable impact on the experimental results. The sensor developed was successful in detecting and quantifying Bcl-2 in the target concentration range.

The sensor can potentially be employed in a point-of-care test device for monitoring and diagnosis at the patient's bedside. The electrical components of the sensing system—RF amplifiers, frequency counter, and analog filter can potentially be miniaturized, assembled, and packaged in a standalone device with the sensor itself. A new, low-cost, accurate, safe, simple, and reliable testing platform to diagnose ovarian cancer by urinary Bcl-2 levels would benefit all women not only in the USA, but worldwide, including medically underserved geographical areas and women at high risk for developing ovarian cancer. This is especially important for detection of early-stage ovarian cancer, which is associated with high survival (>95%) and reduced lifelong medical costs, but currently accounts for less than 10% of diagnosed ovarian cancer cases. In addition to our sensor's ability to accurately detect initial ovarian cancer cases, ovarian cancer monitoring during the course of the disease may indicate recurrent disease and, possibly, therapeutic efficacy. In 2009, 21,550 women were diagnosed with ovarian cancer in the USA [1]; this biosensor could potentially detect thousands of previously-undiagnosed cases. However, it should be noted that the efficacy of the method is limited by biomarker capability/availability. Future work includes plans to collect fresh urine samples from ovarian cancer patients for further validation of the sensor as a detection assay for Bcl-2. The urine samples with concentrations of Bcl-2 characterized by ELISA tests will be tested with this SAW-based sensor.

## Figures and Tables

**Table 1.** Elevated urinary Bcl-2 in cohorts for healthy controls, benign diseases, and early- and late-stage ovarian cancer (N:388) [18].

	**Number of Samples**	**Mean (ng/mL)**	**Std. Dev. (ng/mL)**

**Normal**	77	0.59	0.61
**Benign**	161	1.12	0.79
**Early-Stage Ovarian Cancer**	13	2.60	2.23
**Late-Stage Ovarian Cancer**	137	3.58	1.55

**Table 2.** Sensor design parameters.

Wavelength (λ)	300 μm
Finger width (λ/4)	75 μm
Finger length	6,250 μm
Number of IDT finger pairs	20
Total sensor size	28 mm × 22 mm
Delay path length	12 mm (40λ)
Resonance frequency	16.8 MHz
